# Nutrients, minerals, pigments, phytochemicals, and radical scavenging activity in *Amaranthus blitum* leafy vegetables

**DOI:** 10.1038/s41598-020-59848-w

**Published:** 2020-03-02

**Authors:** Umakanta Sarker, Shinya Oba

**Affiliations:** 1 Department of Genetics and Plant Breeding, Faculty of Agriculture, Bangabandhu Sheikh Mujibur Rahman Agricultural University, Gazipur, 1706 Bangladesh; 2 Laboratory of Field Science, Faculty of Applied Biological Sciences, Gifu University, Yanagido, 1-1 Gifu, Japan

**Keywords:** Biochemistry, Natural variation in plants

## Abstract

*A. blitum* is good sources of abundant natural antioxidant phytopigments such as anthocyanin, betalain, betaxanthin, and betacyanin and antioxidant phytochemicals of interest in the food industry. The chances of utilizing amaranth pigments and phytochemicals had been evaluated for extracting colorful juice as drink purposes. Hence, the presence of nutrients, phytopigments, phytochemicals, and radical scavenging activity of selected *A. blitum* leafy vegetables were evaluated. Leaves of *A. blitum* have considerable fiber, moisture, protein, and carbohydrates. It has considerable magnesium, calcium, potassium (30.42, 24.74, 10.24 mg g^−1^), zinc, iron, copper, manganese, (878.98, 1153.83, 26.13, 207.50 µg g^−1^), phytopigments such as chlorophyll *a*, chlorophyll *ab*, chlorophyll *b*, (63.69, 90.60, 29.32 mg 100 g^−1^), betalain, betaxanthin, betacyanin (112.01, 58.38, 53.63 µg 100 g^−1^), vitamin C (1848.15 µg g^−1^), total carotenoids, β-carotene (1675.38, 1281.66 µg g^−1^), TPC, TFC (253.45 GAE and 162.97 RE µg g^−1^ DW), and TAC (29.46, 55.72 µg g^−1^ DW in Tolax equivalent DPPH and ABTS^+^ radical scavenging capacity) in *A. blitum*. The accessions DS3, DS6, DS8, and DS12 exhibited the highest TAC in Trolox equivalent DPPH and ABTS^+^ radical scavenging capacity, flavonoids, and considerable phytopigments. These accessions had excellent antioxidant profiles along with high yielding potentiality. Hence, *A. blitum* provides an excellent source of proximate, phenolics, minerals, flavonoids, vitamins, and phytopigments to address the nutritional and antioxidant deficiency in daily diet.

## Introduction

The genus *Amaranthus* is C_4_ leafy vegetables of great diversity and plasticity^1^ with many culinary purposes. Bangladesh, Africa, south-east Asia, and South America consumed *A. blitum* as famous leafy vegetables. Its popularity is continuously increasing in the Asian continent and elsewhere because of its attractive leaf color, taste, and adequate nutritional value. In Bangladesh, it can be produced throughout the year as well as in the gaps period of leafy vegetables between winter and hot summer^2,3^. It is very cheap and has adequate protein with essential amino acids, such as methionine and lysine, dietary fiber, minerals, phytopigments, and bioactive compounds, such as betacyanin, chlorophyll, betaxanthin, carotenoids, β-carotene, vitamin C, phenolic compounds, and flavonoids^4–10^.

In the world, food insecurity results in a continuous calorie deficit of approximately 795 million malnourished people^11^. Deficiency of vitamins or minerals results in hidden hunger in over two billion people^12^. Staple foods are deficient of micronutrients, mainly iron, zinc and iodine, pro-vitamin A, carotenoids, vitamin C, E, albeit these are a source of energy^13^. Consequently, staple foods in our daily diet result in hidden hunger^12^. We can ensure a balanced and healthy diet by the consumption of vegetables and fruits as a source of minerals and vitamins accomplished with staple food. Furthermore, we protect human health and decrease the risk of cancer, cardiovascular, and other chronic diseases by consuming vegetables and fruits. Phytochemical compounds, such as phytopigments, vitamin C, phenolics, and flavonoids are thought to contribute to those health benefits^14–16^.

Recently, researchers and consumers interested in natural antioxidants of vegetables. Phytopigments (betacyanin, betaxanthin, chlorophyll, and carotenoids), vitamin C, phenolics and flavonoids are available natural antioxidants in *Amaranths*^4,17^. These natural antioxidant phytochemicals protect many diseases, such as cancer, atherosclerosis, cataracts, cardiovascular diseases, retinopathy, arthritis, emphysema, and neurodegenerative diseases^17–19^. This genus is adapted to abiotic stresses, such as salinity and drought^20–24^.

This species has scarce information albeit it adapted to abiotic stress and low-cost leafy vegetables containing considerable antioxidant phytochemicals, minerals, fiber, and protein. In the previous study, we evaluated *A. tricolor* for morphological, proximate, minerals, antioxidant phytopigments, antioxidant phytochemicals^2,3,5–10^. To our knowledge, it is the first report on phenolics, proximate compositions, phytopigments, mineral, flavonoids, and vitamins in *A. blitum* germplasms. Therefore, this investigation was performed to investigate phenolics, proximate compositions, vitamins, mineral, phytopigments, and flavonoids content in 16 *A. blitum* genotypes and to evaluate variations among traits in 16 *A. blitum* genotypes.

## Results and Discussion

The prominent variations were detected among the studied characters in terms of genotypes.

### Composition of proximate

Fat, moisture, carbohydrates, protein, energy, ash, and dietary fiber contents of *A. blitum* are shown in Table 1. The highest moisture content was recorded in DS13 and DS7 (88.46, 88.45 g 100 g^−1^ FW), while the lowest moisture content was found in DS6 and DS4 (81.43, 81.46 g 100 g^−1^ FW). The range of moisture content was 81.43 to 88.46 g 100 g^−1^ FW. As high dry matter of leaf was obtained from lower moisture contents, seven genotypes (15–18% dry matter) exhibited considerable dry matter. The leaf moisture content of *A. blitum* leafy vegetables directly associated with the maturity of the plant. The findings obtained in this study were corroborated with the results of *A. tricolor* and sweet potato leaves by Sarker and Oba^25^ and Sun *et al*.^26^, respectively.

**Table 1:** Compositions of proximate (per 100 g FW) and dietary fiber (µg g^−1^ FW) of 16 *A. blitum* genotypes.

Genotypes	Moisture (g)	Protein (g)	Fat (g)	Carbohydrates (g)	Energy (kcal)	Ash (g)	Dietary fiber (µg g^−1^ FW)
DS1	85.46 ± 1.87d	1.13 ± 0.03k	0.25 ± 0.03e	9.71 ± 0.07b	46.32 ± 0.35 g	3.45 ± 0.03 g	73.40 ± 1.05 g
DS2	87.82 ± 2.12bc	3.18 ± 0.02 h	0.24 ± 0.02e	5.88 ± 0.08 l	36.05 ± 0.42 l	2.88 ± 0.02j	73.83 ± 1.25 f
DS3	82.74 ± 1.35 f	4.26 ± 0.04d	0.16 ± 0.02 g	7.52 ± 0.07 h	45.93 ± 0.38 g	5.32 ± 0.03b	77.75 ± 0.98e
DS4	81.46 ± 1.51 g	6.22 ± 0.04a	0.18 ± 0.03 f	6.49 ± 0.11j	55.33 ± 0.75b	5.65 ± 0.04a	67.72 ± 0.65j
DS5	88.05 ± 0.76b	2.15 ± 0.03j	0.45 ± 0.02a	6.13 ± 0.14k	35.78 ± 0.62 l	3.22 ± 0.03 h	67.16 ± 0.62k
DS6	81.43 ± 1.17 g	5.45 ± 0.05b	0.28 ± 0.01d	7.72 ± 0.16 g	56.07 ± 0.91a	5.12 ± 0.03d	77.21 ± 0.85e
DS7	88.45 ± 1.33a	4.49 ± 0.03c	0.28 ± 0.02d	1.50 ± 0.07n	26.95 ± 0.72 m	5.28 ± 0.05c	68.80 ± 0.88 h
DS8	82.76 ± 1.62 f	3.55 ± 0.03 f	0.14 ± 0.03 h	9.00 ± 0.15d	49.75 ± 0.53d	4.55 ± 0.01e	91.94 ± 0.52d
DS9	86.49 ± 1.24c	3.38 ± 0.04 g	0.34 ± 0.01c	6.57 ± 0.12j	40.27 ± 0.49i	3.22 ± 0.01 h	59.96 ± 0.75 m
DS10	84.65 ± 1.18e	2.26 ± 0.04i	0.29 ± 0.02d	8.56 ± 0.17e	43.74 ± 0.62 h	4.24 ± 0.06 f	66.54 ± 0.62 l
DS11	88.07 ± 1.74b	3.39 ± 0.02 g	0.32 ± 0.02 cd	6.17 ± 0.16k	39.78 ± 0.4j3	2.05 ± 0.02 l	68.66 ± 0.47i
DS12	84.73 ± 1.64e	3.59 ± 0.06 f	0.29 ± 0.01d	8.33 ± 0.11 f	48.00 ± 0.48e	3.06 ± 0.02i	95.65 ± 0.35b
DS13	88.46 ± 1.87a	4.26 ± 0.06d	0.41 ± 0.04b	4.59 ± 0.13 m	36.93 ± 0.46k	2.28 ± 0.03k	97.88 ± 0.62a
DS14	84.95 ± 1.05e	2.29 ± 0.05i	0.33 ± 0.03c	9.21 ± 0.12c	47.51 ± 0.65 f	3.22 ± 0.05 h	69.15 ± 0.53 h
DS15	82.54 ± 1.13 f	2.86 ± 0.05i	0.18 ± 0.01 f	10.17 ± 0.13a	52.85 ± 0.51c	4.25 ± 0.06 f	92.35 ± 0.42c
DS16	86.43 ± 1.62c	3.66 ± 0.04e	0.34 ± 0.01c	6.69 ± 0.11i	43.95 ± 0.67 h	2.88 ± 0.02j	77.63 ± 0.48e
Mean	85.28	3.51	0.28	7.14	44.08	3.79	76.60
CV%	1.060	0.132	0.015	0.135	0.226	0.564	0.419

FW, fresh weight, CV, Coefficient of variation; n = 3; **Significant at 1% level, Different letters in each columns are differed significantly by Tukey’s HSD test.

Leaves of *A. blitum* exhibited pronounced variability in terms of protein compositions. The accession DS4 showed the highest content of protein (6.22 g 100 g^−1^), while the lowest content of protein was obtained from the genotype DS1 (1.13 g 100 g^−1^). Eleven accessions had greater content of protein compared to their average values. As leafy vegetables, the accessions DS4, SA6, DS7, DS3, and DS13 had high protein content. *A. blitum* leafy vegetables are the main source of protein for poor people of the low-income countries and vegetarians. It revealed that the protein content of *A. blitum* (3.51 g 100 g^−1^) was higher than *A. tricolor* (1.26%) of our previous study^2^.

The content of fat was the highest in DS5 (0.45 g 100 g^−1^ FW) showing the order: DS13 > DS9 > DS14 > DS11. The lowest fat content was found in DS8 (0.14 g 100 g^−1^ FW) with a grand mean value of 0.28 g 100 g^−1^ FW. Sarker and Oba^25^ and Sun *et al*.^26^ observed similar results in *A. tricolor* and the leaves of sweet potato, respectively, They reported that cell function, the insulation of body organs, and body temperature were maintained through catabolism of fat. Fats are an excellent source of fatty acids containing omega-6 and omega-3. Absorption, transport, and digestion of fat-soluble vitamins, such as E, K, A, and D principally depend on fats. The genotype DS15 had the highest carbohydrates content (10.17 g 100 g^−1^ FW) followed by DS1, DS14, and DS8 while the lowest carbohydrates content was noted in DS7 (1.50 g 100 g^−1^) with a mean value of 7.14 g 100 g^−1^ FW. The highest energy was recorded in the accession DS6 (56.07 kcal 100 g^−1^) followed by DS4, DS15, DS8, and DS12, while the accession DS7 exhibited the lowest energy content (26.95 kcal 100 g^−1^) with a grand mean value of 44.08 kcal 100 g^−1^. The highest ash content was noted in DS4 (5.65 g 100 g^−1^) followed by DS3, DS7, DS6, and DS8, while ash content was the lowest in DS11 (2.05 g 100 g^−1^) with a grand mean value of 3.79 g 100 g^−1^.

The considerable variations were observed in 16 *A. blitum* genotypes in terms of dietary fiber. The accession DS13 showed the highest content of fiber (97.88 µg g^−1^) followed by DS12, DS15, and DS8 whereas the lowest content of fiber was noted in DS9 (59.96 µg g^−1^) with a mean value of 76.60 µg g^−1^. Dietary fiber significantly contributed to the cure of constipation, digestibility, and palatability^6^. Our results exhibited that the leaves of *A. blitum* were a considerable amount of dietary fiber, moisture, carbohydrates, and protein. The results of this study corroborated with the results of Sarker and oba^25^. The genotype DS4 could be used as dry matter, protein, and ash enrich leafy vegetables. The genotype DS15 could be used as carbohydrates enrich leafy vegetables, while The genotype DS13 could be used as dietary fiber and DS6 as calories enrich leafy vegetables.

### Composition of minerals

Manganese, potassium, copper, magnesium, iron, calcium, and zinc content of *A. blitum* are shown in Table 2. In this study, the range of potassium content was 0.95 to 16.28 mg g^−1^. The accessions DS2, DS4, DS14, DS12, DS15, DS5, and DS1 showed good content of potassium, while the lowest potassium content was reported in the accession DS10, with mean potassium content of 10.24 mg g^−1^. The potassium content of nine genotypes was much higher than their grand mean. The range of calcium content was 15.22–32.82 mg g^−1^ DW. The accessions DS1, DS7, DS14, DS10, DS4, DS11, and DS13 had good calcium content, while the lowest calcium content was recorded in the accession DS16 with a mean calcium content of 24.74 mg g^−1^. High calcium content was noted in seven accessions which were better than the respective average value. The accession DS2 had the highest magnesium content. In contrast, the accessions DS8, DS9, and DS16 showed the lowest magnesium content with a mean value of 30.42 mg g^−1^. The accessions DS2, DS5, DS1, DS13, DS6, DS7, DS10, and DS12 had considerable magnesium content. Magnesium content did not exhibit pronounced variations in 16 *A. blitum* genotypes (28.63 to 35.43 mg g^−1^). Our study revealed that we noted a considerable amount of potassium (10.24 mg g^−1^), calcium (24.74 mg g^−1^) and magnesium (30.42 mg g^−1^) in the leaf of *A. blitum*, albeit we determined based on the dry weight. Chakrabarty *et al*.^27^ in stem amaranth and Sarker and Oba^25^ in *A. tricolor* also observed similar results. Jimenez-Aguiar and Grusak^28^ reported a good amount of Mg, K, and Ca in different species of amaranth. They reported that Mg, Ca, and K content of different species of amaranth was much greater than kale, black nightshade, spider flower, and spinach.

**Table 2:** Composition of Minerals (Macro and microelements, µg g^−1^ DW and mg g^−1^ DW, respectively) of 16 *A. blitum* genotypes.

Genotypes	Macro elements (mg g^−1^ DW)			Micro elements (µg g^−1^ DW)			
	Potassium	Calcium	Magnesium	Iron	Manganese	Copper	Zinc
DS1	11.39 ± 0.05 f	32.82 ± 0.03a	31.13 ± 0.09c	881.62 ± 0.75 m	264.09 ± 0.42b	18.19 ± 0.04j	720.05 ± 0.56j
DS2	16.28 ± 0.05a	22.07 ± 0.02 h	35.43 ± 0.12a	1525.30 ± 0.92b	356.84 ± 0.15a	27.25 ± 0.03 f	1473.54 ± 0.42a
DS3	8.89 ± 0.07j	24.02 ± 0.01 f	30.19 ± 0.14e	1035.49 ± 0.47j	196.48 ± 0.51 f	45.12 ± 0.02a	1082.09 ± 0.35c
DS4	13.86 ± 0.04b	19.22 ± 0.06j	29.26 ± 0.17 h	1118.4 ± 0.53 h	152.76 ± 0.24k	12.09 ± 0.06k	652.63 ± 0.62 m
DS5	11.63 ± 0.02e	27.15 ± 0.02e	32.05 ± 0.21b	1131.32 ± 0.75 g	251.35 ± 0.23c	27.88 ± 0.08e	980.26 ± 0.64e
DS6	10.62 ± 0.07 g	24.02 ± 0.09 f	30.51 ± 0.21d	2057.02 ± 0.51a	132.65 ± 0.42 l	18.06 ± 0.05j	601.37 ± 0.47n
DS7	8.94 ± 0.03j	32.02 ± 0.06b	30.51 ± 0.14d	902.63 ± 0.27 l	241.15 ± 0.65e	38.05 ± 0.04b	1200.24 ± 0.45b
DS8	10.46 ± 0.06 h	16.82 ± 0.06k	28.63 ± 0.14i	1097.61 ± 0.17i	176.21 ± 0.62i	20.10 ± 0.05i	681.38 ± 0.85 l
DS9	6.55 ± 0.07 l	23.22 ± 0.05 g	28.63 ± 0.18i	1375.91 ± 0.37d	245.90 ± 0.46d	34.09 ± 0.06c	950.14 ± 0.48 g
DS10	0.95 ± 0.05 m	30.42 ± 0.06d	30.51 ± 0.17d	1117.46 ± 0.75 h	196.62 ± 0.64 f	28.25 ± 0.06d	680.41 ± 0.52 l
DS11	8.65 ± 0.06k	27.22 ± 0.07e	29.88 ± 0.12 f	195.12 ± 0.43n	197.38 ± 0.85 f	24.15 ± 0.05 g	961.21 ± 0.64 f
DS12	12.06 ± 0.05d	20.02 ± 0.08i	30.51 ± 0.19d	1431.73 ± 0.24c	157.43 ± 0.68j	22.15 ± 0.03 h	700.27 ± 0.46k
DS13	9.83 ± 0.05i	27.22 ± 0.04e	31.13 ± 0.12c	1176.18 ± 0.46 f	177.45 ± 0.56i	38.16 ± 0.04b	681.07 ± 0.53 l
DS14	12.27 ± 0.04c	31.22 ± 0.08c	29.57 ± 0.18 g	1096.57 ± 0.53i	183.07 ± 0.92 h	24.19 ± 0.06 g	986.79 ± 0.54d
DS15	11.51 ± 0.03e	23.22 ± 0.07 g	30.19 ± 0.14e	983.33 ± 0.57k	193.49 ± 0.83 g	22.23 ± 0.04 h	851.75 ± 0.62i
DS16	9.88 ± 0.06i	15.22 ± 0.04 l	28.63 ± 0.12i	1335.65 ± 0.82e	197.14 ± 0.52 f	18.05 ± 0.07j	860.51 ± 0.62 h
Mean	10.24	24.74	30.42	1153.83	207.50	26.13	878.98
CV%	2.075	1.280	1.329	0.3574	0.6706	0.3287	0.1348

CV, Coefficient of variation; n = 3; **Significant at 1% level, Different letters in each columns are differed significantly by Tukey’s HSD test.

Iron content showed prominent variations in terms of genotypes (195.12 to 2057.02 µg g^−1^). The highest iron content was observed in the genotypes DS6. In contrast, the lowest iron content was obtained from the genotype DS11, with a mean iron content of 1153.83 µg g^−1^. Six accessions exhibited higher content of iron than their mean iron content. The range of manganese content was 132.65 to 356.84 µg g^−1^, with a mean value of 207.50 µg g^−1^. The accessions DS2, DS1, DS5, DS9, and DS7 had considerable content of manganese, while the lowest manganese content was recorded in the genotype DS6 (132.65 µg g^−1^). Copper content exhibited considerable variations in terms of accessions (16.09–45.12 µg g^−1^). The accession DS3 showed the highest copper content (45.12 µg g^−1^), followed by DS7, DS9, DS10, DS5, and DS2. Seven accessions showed better copper content than the average value (26.13 µg g^−1^). The accession varied considerably in the content of zinc (680.41, 681.07, 681.38 µg g^−1^ in DS10, DS13, and DS8, respectively to 1473.54 µg g^−1^ in DS2). High zinc content was observed in seven genotypes which were higher than the grand mean value (878.98 µg g^−1^). Three genotypes DS2, DS3, and DS7 exhibited excellent zinc content (1082.09 to 1473.54 µg g^−1^ DW). Leaves of *A. blitum* contained higher zinc and iron compared to beach pea^29^ and the leaves of cassava^30^. Our study showed that leaves of *A. blitum* had considerable iron (1153.83 µg g^−1^), manganese (207.50 µg g^−1^), copper (26.13 µg g^−1^), and zinc (878.98 µg g^−1^), albeit it was measured based on the dry weight. Jimenez-Aguiar and Grusak^28^ reported a good amount of iron, manganese, copper, and zinc in the different species of amaranth. They reported that iron, manganese, copper, and zinc content of different species of amaranth were much greater than kale, black nightshade, spider flower, and spinach. The genotype DS2 could be used as potassium, magnesium, iron, manganese, and zinc enrich leafy vegetables. The genotype DS1 could be used as calcium enrich leafy vegetable, while DS3 could be used as copper and DS6 as iron enrich leafy vegetables.

### Composition of antioxidant phytopigments

Table 3 represents the composition of antioxidant phytopigments of 16 *A. blitum* genotypes. Chlorophyll *a* content differed remarkably in *A. blitum* genotypes (13.17 to 63.69 mg 100 g^−1^). The highest chlorophyll *a* content was obtained from the genotype DS8 (63.69 mg 100 g^−1^), while the accessions DS3 and DS2 showed the lowest chlorophyll *a* (13.17 and 13.26 mg 100 g^−1^). Chlorophyll *a* content was high in the genotypes DS13, DS4, DS6, and DS15. The chlorophyll *a* content of six genotypes was higher than the average value. There were prominent variations in chlorophyll *b* content of 16 *A. blitum* genotypes (4.97 to 29.32 mg 100 g^−1^). The highest chlorophyll *b* content was observed in DS15 (29.32 mg kg^−1^), followed by DS8, DS4, DS12, and DS6. Conversely, DS7 had the lowest chlorophyll *b* (4.97 mg 100 g^−1^). Prominent variations were also observed in chlorophyll *ab* (19.72 to 90.60 mg 100 g^−1^). DS8, DS4, DS13, DS15, and DS6 showed good content of chlorophyll *ab*, while DS2 had the lowest chlorophyll *ab* content (19.72 mg 100 g^−1^). Eight accessions exhibited higher content of chlorophyll *ab* than the mean chlorophyll *ab* content. Our study revealed that stem amaranth genotypes had a considerable amount of chlorophyll *ab* (90.60 mg 100 g^−1^), chlorophyll *a* (63.69 mg 100 g^−1^), and chlorophyll *b* (29.32 mg 100 g^−1^), whereas, chlorophylls content of *A. tricolor* reported by Khanam and Oba^31^ were relatively lower.

**Table 3:** Performance of antioxidant phytopigments in 16 *A. blitum* genotypes.

Genotypes	chlorophyll *a* (mg 100 g^−1^ FW)	Chlorophyll *b* (mg 100 g^−1^ FW)	Chlorophyll *ab* (mg 100 g^−1^ FW)	Betacyanin (µg 100 g^−1^ FW)	Betaxanthin (µg 100 g^−1^ FW)	Betalain (µg 100 g^−1^ FW)	Total carotenoids (µg g^−1^ FW)
DS1	17.55 ± 0.05 l	8.36 ± 0.04 l	25.94 ± 0.14 m	28.58 ± 0.12 h	25.53 ± 0.18i	54.13 ± 0.31i	1050.55 ± 1.25 g
DS2	13.26 ± 0.06n	6.44 ± 0.06n	19.72 ± 0.12p	18.45 ± 0.14n	18.50 ± 0.19 m	36.96 ± 0.25n	792. 51 ± 1.34k
DS3	13.1 7 ± 0.07n	7.23 ± 0.06 m	20.42 ± 0.16o	23.59 ± 0.16k	23.46 ± 0.18j	47.07 ± 0.16k	679.84 ± 0.58 m
DS4	51.52 ± 0.06c	25.15 ± 0.09c	76.69 ± 0.14b	50.35 ± 0.13b	50.37 ± 0.21b	100.73 ± 0.26b	516.80 ± 0.61o
DS5	28.43 ± 0.07 h	13.73 ± 0.08i	42.51 ± 0.15i	29.51 ± 0.42 g	30.02 ± 0.25 g	59.86 ± 0.51 g	1112.31 ± 1.25e
DS6	44.15 ± 0.05d	21.88 ± 0.06e	66.06 ± 0.12e	45.26 ± 0.22c	46.53 ± 0.24c	91.80 ± 0.48c	1080.85 ± 1.36 f
DS7	17.25 ± 0.05 m	4.97 ± 0.06p	22.24 ± 0.18n	10.54 ± 0.14o	9.69 ± 0.18n	20.24 ± 0.43o	1592.06 ± 1.64b
DS8	63.69 ± 0.09a	26.88 ± 0.03b	90.60 ± 0.14a	40.60 ± 0.14e	42.58 ± 0.15d	83.19 ± 0.18e	742.84 ± 1.46 l
DS9	23.90 ± 0.08i	12.75 ± 0.08j	36.67 ± 0.12j	20.90 ± 0.18 m	22.27 ± 0.21k	43.18 ± 0.28 m	1175.03 ± 1.64d
DS10	20.84 ± 0.07j	5.56 ± 0.08o	26.43 ± 0.18 l	28.26 ± 0.25i	29.57 ± 0.26 h	57.84 ± 0.35 h	1305.34 ± 1.28c
DS11	30.59 ± 0.07 g	17.46 ± 0.08 h	48.08 ± 0.16 h	29.43 ± 0.21 g	25.51 ± 0.15i	54.96 ± 0.16i	905.99 ± 1.14 h
DS12	30.49 ± 0.03 g	22.82 ± 0.03d	53.33 ± 0.24 g	53.63 ± 0.51a	58.38 ± 0.23a	112.01 ± 0.43a	1005.79 ± 1.34
DS13	52.37 ± 0.05b	17.85 ± 0.06 g	70.25 ± 0.18c	30.52 ± 0.31 f	30.87 ± 0.27 f	61.41 ± 0.28 f	907.35 ± 1.32j
DS14	20.65 ± 0.07k	9.19 ± 0.06k	29.87 ± 0.16k	25.43 ± 0.27j	25.57 ± 0.41i	51.01 ± 0.18j	1675.38 ± 1.34a
DS15	40.52 ± 0.06e	29.32 ± 0.06a	69.86 ± 0.12d	22.46 ± 0.19 l	21.77 ± 0.15 l	44.25 ± 0.46 l	936.89 ± 1.35i
DS16	38.24 ± 0.06 f	19.47 ± 0.08 f	57.73 ± 0.16 f	42.67 ± 0.46d	41.56 ± 0.18e	84.26 ± 0.35d	588.03 ± 1.18n
Mean	32.90	15.57	47.28	31.26	31.39	62.68	1018.34
CV%	4.143	2.123	3.214	3.342	2.164	3.253	5.622

CV, Coefficient of variation; n = 3; **Significant at 1% level, Different letters in each columns are differed significantly by Tukey’s HSD test.

Betacyanin ranged from 10.54 to 53.63 µg 100 g^−1^ with a mean betacyanin content of 31.26 µg 100 g^−1^. DS12 exhibited the highest betacyanin content (53.63 µg 100 g^−1^), followed by DS4, DS6, DS16, and DS8. In contrast, DS7 exhibited the lowest betacyanin (10.54 µg 100 g^−1^). Betaxanthin content showed the significant and notable differences in 16 *A. blitum* genotypes (18.50 to 58.38 µg 100 g^−1^). DS12 had the highest betaxanthin content (58.38 µg g^−1^). High betaxanthin content was recorded in DS4, DS6, DS8, and DS16 with the lowest betaxanthin content of 18.50 µg 100 g^−1^ in DS2. Five accessions had higher betaxanthin content than the mean value. Pronounced variations were observed in betalain content (20.24 to 112.01 µg 100 g^−1^). DS12 had the highest betalain (112.01 µg 100 g^−1^), and DS4, DS6, DS16, and DS8 exhibited high betalain content. In contrast, the accession DS7 showed the lowest content of betalain (20.24 µg 100 g^−1^). Five genotypes had higher betalain content than average value. The range of total carotenoids content was 516.80 µg g^−1^ in DS4 to 1675.38 µg g^−1^ in DS14. DS14 showed the highest total carotenoids content (1675.38 µg g^−1^) and DS7, DS10, DS9, and DS5 showed good total carotenoids content. Seven accessions had higher total carotenoids than average value. In this study, we found considerable betacyanin (53.63 µg 100 g^−1^), betaxanthin (58.38 µg 100 g^−1^), betalain (112.01 µg 100 g^−1^) and total carotenoids (1675.38 µg g^−1^) in *A. blitum*, which corroborated with the results of Khanam *et al*.^32^ for betacyanin, betaxanthin, betalain, and total carotenoids content of *A. tricolor*. The genotype DS8 could be used as chlorophylls enrich leafy vegetable. The genotype DS12 could be used as betacyanin, betaxanthin and belatain enrich leafy vegetables, while the genotypes DS14 could be used as total carotenoids enrich leafy vegetables.

### Antioxidant phytochemicals

Table 4 represents TAC, vitamins, TPC, and TFC of *A. blitum*. The range of β-carotene content was 441.85 µg g^−1^ in DS16 to 1281.66 µg g^−1^ in DS14. The highest β-carotene content was exhibited in DS14 (1281.66 µg g^−1^) and DS7, DS10, DS9, and DS5 showed high β-carotene content. Seven accessions had higher β-carotene than average β-carotene content. The range of vitamin C content was 123.19 µg g^−1^ in the genotype DS12 to 1848.15 µg g^−1^ in the genotype DS7, with a mean value of 928.83 µg g^−1^. Five accessions showed higher vitamin C than the average content of vitamin C. Content of vitamin C was excellent (more than 1100 µg g^−1^) in four genotypes DS7, DS4, DS1, and DS15. The range of total polyphenol content (TPC) was 92.33 GAE µg g^−1^ (DS2) to 253.45 GAE µg g^−1^ (DS4) with an average TPC content of 175.83 GAE µg g^−1^. DS4 showed the highest total polyphenol content. DS13, DS8, DS16, and DS6 showed high total polyphenol content values. Nine accessions showed higher polyphenol than average polyphenol content. Prominent variations were noted in the TFC content of *A. blitum* genotypes, with a range of 65.23 RE µg g^−1^ in the accession DS1 to 162.97 RE µg g^−1^ in the accession DS3. The average value of the total flavonoids content was 119.45 RE µg g^−1^ DW. DS3 exhibited the highest TFC showing the order: DS3 ˃ DS8 ˃ DS4 ˃ DS12 ˃ DS13 ˃ DS16. Nine accessions showed higher TFC values than average TFC. The range of TAC in Trolox equivalent DPPH radical scavenging capacity was 12.27 µg g^−1^ (DS2) to 29.46 µg g^−1^ (DS6, DS8, and DS12). Three genotypes DS12, DS8, and DS6 had the highest TAC in Trolox equivalent DPPH radical scavenging capacity. The accessions DS3, DS13, DS16, and DS4 showed high TAC in Trolox equivalent DPPH radical scavenging capacity. In contrast, DS2 had the lowest TAC in Trolox equivalent DPPH radical scavenging capacity with mean TAC of 20.90 TEAC µg g^−1^ DW. Seven accessions exhibited higher TAC in Trolox equivalent DPPH radical scavenging capacity than average value. The range of TAC in Trolox equivalent ABTS^+^ radical scavenging capacity was 21.93 µg g^−1^ (DS2) to 55.72 µg g^−1^ (DS6). The accessions DS6, DS3, DS12, and DS8 exhibited the highest TAC in Trolox equivalent ABTS^+^ radical scavenging capacity (55.72, 55.65. 55.62, 55.04 µg g^−1^). High TAC in Trolox equivalent ABTS^+^ radical scavenging capacity was noticed in the accessions, DS13, DS16, DS4, and DS7. Conversely, the lowest TAC in Trolox equivalent ABTS^+^ radical scavenging capacity was observed in DS2 with an average of 21.93 TEAC µg g^−1^ DW. Seven accessions had higher TAC in Trolox equivalent ABTS^+^ radical scavenging capacity than average TAC in Trolox equivalent ABTS^+^ radical scavenging capacity.

**Table 4:** Performance of TPC, β-carotene, TAC (ABTS^+^ and DPPH), vitamin C, and TFC of 16 *A. blitum* genotypes.

Genotypes	β-carotene (µg g^−1^ FW)	Vitamin C (µg g^−1^ FW)	TPC (GAE µg g^−1^ DW)	TFC (RE µg g^−1^ DW)	TAC (DPPH) (TEAC µg g^−1^ DW)	TAC (ABTS^+^) (TEAC µg g^−1^ DW)
DS1	800.42 ± 1.52 g	1722.38 ± 2.31c	122.16 ± 0.33 m	65.23 ± 0.23 l	15.25 ± 0.10i	28.75 ± 0.05j
DS2	601.15 ± 1.34 l	800.65 ± 3.10 h	92.33 ± 0.25o	98.81 ± 0.22i	12.27 ± 0.09 l	21.93 ± 0.08n
DS3	516.84 ± 1.52n	985.72 ± 2.05e	198.75 ± 0.26i	162.97 ± 0.15a	28.65 ± 0.21b	55.65 ± 0.04a
DS4	392.24 ± 1.28p	1786.76 ± 2.85b	253.45 ± 0.36a	145.25 ± 0.23c	24.31 ± 0.24e	46.44 ± 0.06e
DS5	844.34 ± 1.18e	755.41 ± 2.09i	146.87 ± 0.42j	102.82 ± 0.17 g	16.51 ± 0.22 g	31.86 ± 0.08 h
DS6	823.46 ± 1.36 f	616.04 ± 1.56k	219.55 ± 0.46e	145.25 ± 0.28c	29.46 ± 0.24a	55.72 ± 0.03a
DS7	1208.41 ± 1.65b	1848.15 ± 1.69a	201.53 ± 0.25 h	102.27 ± 0.34 g	20.14 ± 0.33 f	38.64 ± 0.06 f
DS8	564.14 ± 1.86 m	924.29 ± 1.58 f	246.20 ± 0.12c	155.34 ± 0.32b	29.46 ± 0.18a	55.04 ± 0.08a
DS9	901.16 ± 1.33d	802.06 ± 2.78 h	98.74 ± 0.34n	97.84 ± 0.15j	15.17 ± 0.22j	29.35 ± 0.04i
DS10	991.13 ± 2.84c	862.35 ± 1.65 g	92.54 ± 0.42o	82.47 ± 0.32k	12.83 ± 0.19k	22.98 ± 0.04 m
DS11	691.25 ± 1.52j	492.84 ± 1.34 l	201.90 ± 0.29 g	100.97 ± 0.16 h	16.28 ± 0.21 h	33.40 ± 0.06 g
DS12	763.34 ± 1.54 h	123.19 ± 2.36n	213.78 ± 0.13 f	135.54 ± 0.16d	29.46 ± 0.19a	55.62 ± 0.06a
DS13	684.68 ± 1.78k	924.26 ± 2.36 f	246.46 ± 0.15b	135.66 ± 0.35d	28.31 ± 0.18c	50.91 ± 0.08c
DS14	1281.66 ± 1.29a	431.12 ± 2.46 m	125.28 ± 0.18 l	125.64 ± 0.23 f	14.55 ± 0.18j	26.19 ± 0.05 l
DS15	711.70 ± 2.30i	1108.46 ± 1.25d	128.29 ± 0.62k	125.27 ± 0.28 f	15.26 ± 0.14i	28.52 ± 0.08k
DS16	441.85 ± 1.56o	677.58 ± 3.14j	225.42 ± 0.52d	129.91 ± 0.28e	26.48 ± 0.19d	48.49 ± 0.08d
Mean	763.61	928.83	175.83	119.45	20.90	39.30
CV%	4.853	1.427	2.126	0.342	0.1234	0.2456

CV, Coefficient of variation; TAC = Total antioxidant capacity, TPC = Total polyphenol content, TFC = Total flavonoid content, n = 3; **Significant at 1% level, Different letters in each columns are differed significantly by Tukey’s HSD test.

In this study, we reported considerable β-carotene (1281.66 µg g^−1^) and vitamin C (1848.15 µg g^−1^) in *A. blitum*, which was relatively higher than *A. tricolor*^3^ of our earlier studies. Our obtained TPC (253.45 GAE µg g^−1^ FW) was higher than the TPC of *A. tricolor* reported by Khanam *et al*.^32^. Our reported TFC (162.97 RE µg g^−1^ DW) and TAC (ABTS^+^ and DPPH) (55.72 and 29.46 TEAC µg g^−1^ DW) were corroborative to the results of *A. tricolor* of Khanam *et al*.^32^. The accessions DS14, DS7, and DS4 could be used as beta-carotene, vitamin C, and TPC enrich leafy vegetables, respectively. The accession DS3 showed the highest TAC (ABTS^+^ and DPPH), flavonoids, and copper, as well as DS6, exhibited the highest TAC (ABTS^+^ and DPPH), flavonoids, and iron. Similarly, The accession DS8 contained the highest TAC (ABTS^+^ and DPPH), chlorophylls, flavonoids, and polyphenols, as well as DS12, showed the highest TAC (ABTS^+^ and DPPH), flavonoids, betacyanin, betalain, and betaxanthin. These four accessions had excellent antioxidant profiles along with high yielding potentiality. Hence, *A. blitum* provides an excellent source of proximate, phenolics, minerals, flavonoids, vitamins, and phytopigments to address the nutritional and antioxidant deficiency in daily diet.

### Correlation studies

The coefficient of **c**orrelation of biologically active compounds of *A. blitum* is shown in Table 5. The coefficient of **c**orrelation of biologically active compounds shown in Table 5 had interesting results. We observed a significant positive correlation among TAC (DPPH), chlorophyll *ab*, betacyanin, chlorophyll *a*, betaxanthin, betalain, TAC (ABTS^+^), chlorophyll *b*, and TFC. Shukla *et al*.^33^ also reported positive associations in their earlier work in *A. tricolor*. Similarly, betacyanin, betaxanthin, and betalain showed positive and significant interrelationships among each of them and with TAC (ABTS^+^), chlorophylls, TFC, TAC (DPPH), and TPC which was corroborated with the results of our earlier studies^8,9^ indicating an increase in any phytopigment was directly related to increment of another phytopigment. The positive and significant interrelationships of TAC (DPPH), all phytopigments, TAC (ABTS^+^), TFC, and TPC indicated that phytopigments, TFC, and TPC exhibited strong antioxidant potential. The significant negative association was observed between phytopigments *vs*. total carotenoids and phytopigments *vs*. beta-carotene, while total carotenoids and beta-carotene exhibited a significant positive association with TAC (ABTS^+^), TAC (DPPH), TPC, and TFC which was corroborated with the results of our earlier studies in amaranth^20–24^. It indicated that the increment of any phytopigment had a direct decrement of total carotenoids and beta-carotene. The positive and significant interrelationship of total carotenoids and beta-carotene with TPC, TAC (ABTS^+^ and DPPH), and TFC signifies that β-carotene and total carotenoids had excellent antioxidant potentiality. There were positive associations between beta-carotene and total carotenoids. In contrast, the negligible insignificant association was observed between vitamin C and all the characters indicating that vitamin C had no contribution to the antioxidant activity of *A. blitum*. Jimenez-Aguilar and Grusak^28^ reported a negligible insignificant association for ascorbic acid in amaranth. The positive and significant associations were observed among TAC (ABTS^+^), TPC, TAC (DPPH), and TFC as well as all phytopigments, and vitamins indicating the contribution of these compounds in the antioxidant potentiality of *A. blitum* genotypes. Our reported results revealed that phytopigments, vitamins, phenolics, and total flavonoids played a significant contribution to the antioxidant capacity of *A. blitum*.

**Table 5:** Coefficient of correlation for antioxidant phytopigments, β-carotene, TAC (ABTS^+^ and DPPH), vitamin C, TPC, and TFC in 16 *A. blitum* genotypes.

Traits	Chl *b* (mg 100 g^−1^ FW)	Chl *ab* (mg 100 g^−1^ FW)	Beta cyanin (µg 100 g^−1^ FW)	Beta xanthin (µg 100 g^−1^ FW)	Betalain (µg 100 g^−1^ FW)	Total catonenoirds (µg g^−1^ FW)	β- carotene (µg g^−1^ FW)	Vitamin C (µg g^−1^ FW)	TPC (GAE µg g^−1^ DW)	TFC (RE µg g^−1^ DW)	TAC (TEAC µg g^−1^ DW)	TAC (ABTS^+^) (TEAC µg g^−1^ DW)
Chlorophyll *a* (mg100 g^−1^ FW)	0.88**	0.78**	0.62**	0.61**	0.61**	−0.53**	−0.44**	−0.001	0.45**	0.44**	0.55**	0.63**
Chlorophyll *b* (mg 100 g^−1^ FW)		0.83**	0.60**	0.58**	0.59**	−0.55**	−0.49**	−0.011	0.44**	0.45**	0.53**	0.57**
Chlorophyll *ab* (mg 100 g^−1^ FW)			0.64**	0.62**	0.63**	−0.62**	−0.48**	−0.007	0.47**	0.47**	0.57**	0.53**
Betacyanin (µg 100 g^−1^ FW)				0.85**	0.87**	−0.71**	−0.54**	−0.10	0.53**	0.55**	0.61**	0.68**
Betaxanthin (µg 100 g^−1^ FW)					0.85**	−0.66**	−0.52**	−0.12	0.51**	0.54**	0.60**	0.68**
Betalain (µg 100 g^−1^ FW)						−0.67**	−0.53**	−0.11	0.52**	0.54**	0.61**	0.75**
Total catonenoirds (µg g^−1^ FW)							0.84**	−0.18	0.54**	0.48**	0.68**	0.85**
β-carotene (µg g^−1^ FW)								−0.17	0.29*	0.44**	0.57**	0.54**
Vitamin C (µg g^−1^ FW)									0.05	0.02	0.06	0.08
TPC (GAE µg g^−1^ DW)										0.74**	0.66**	0.95**
TFC (RE µg g^−1^ DW)											0.74**	0.79**
TAC (DPPH) (TEAC µg g^−1^ DW)												0.98**

Chl *a*, Chlorophyll *a*; Chl *ab*, Chlorophyl *ab*; TAC, Total antioxidant capacity; TPC, Total polyphenol content; TFC, Total flavonoid content; *Significant at 5% level; **Significant at 1% level.

In conclusion, *A. blitum* leaves were good sources of K, Ca, Mg, iron, manganese, copper, zinc, chlorophyll, vitamin C, betacyanin, betaxanthin, TAC, betalain, carotenoids, β-carotene, dietary fiber, carbohydrates, protein, TPC, and TFC. It could be used as leafy vegetables for potential sources of antioxidant phytopigments, vitamin C, β-carotene, phenolics, minerals and proximate, flavonoids in the human diet to address the nutritional deficiency and gaining antioxidant and nutritional sufficiency. Details studies on animal models and humans are prerequisites to confirm nutrition and pharmacology before promoting the use of the leaves for health purposes.

## Methods

### Experimental design, layout, materials, and cultural practices

Sixteen *A. blitum* accessions selected from 75 genotypes were sown in Bangabandhu Sheikh Mujibur Rahman Agricultural University, Gazipur, in a randomized complete block design (RCBD) with three replicates. The experimental unit was 1 m × 1 m. The amaranth genotypes were grown maintaining the distance of 20 cm between rows and 5 cm between plants. During land preparation, total compost (10 ton/ha) was applied. Appropriate fertilizer doses, such as triple superphosphate, urea, gypsum, and murate of potash at 100, 200, 30, and 150 kg/ha, respectively were maintained. We maintained appropriate spacing between plants of a row through necessary thinning. Weeding and hoeing were done to remove the weeds. Adequate irrigations were applied to ensure the normal growth of amaranth. Leaves were harvested at 30 days old.

### Solvents and reagents

Solvents: methanol, acetone, and ethanol. Reagents: NaOH, dithiothreitol (DTT), HNO_3_, standard compounds of pure Trolox (6-hydroxy-2, 5, 7, 8-tetramethyl-chroman-2-carboxylic acid), cesium chloride, ascorbic acid, H_2_O_2,_ H_2_SO_4_, potassium persulfate, ascorbic acid, HClO_3,_ folin-ciocalteu reagent, gallic acid, DPPH (2,2-diphenyl1-picrylhydrazyl), ABTS^+^ (2,2-azino-bis-(3-ethylbenzothiazoline-6-sulfonic acid), rutin, 2,2-dipyridyl, sodium carbonate, aluminum chloride hexahydrate, and potassium acetate. We bought all solvents and reagents from Kanto Chemical Co. Inc. (Tokyo, Japan) and Merck (Germany).

### Measurement of the composition of proximate

Ash, crude fat, moisture, crude protein contents, fiber, and gross energy were determined following AOAC method^34,35^. Crude protein was estimated through the Micro-Kjeldahl method multiplying nitrogen by 6.25 (AOAC method 976.05). To estimate carbohydrate (g 100 g^−1^ FW), the total percentage of protein, ash, fat, and moisture was subtracted from 100.

### Estimation of composition of minerals

*A. blitum* leaf samples were dried for 24 hours at 70 °C in an oven. We ground the dried leaves in a mill finely. Calcium, potassium, magnesium, iron, manganese, copper, and zinc were determined following nitric-perchloric acid digestion method^36^. Exactly 0.5 g dried leaf sample was digested with 40 ml HClO_4_ (70%), 400 ml HNO_3_ (65%), and 10 ml H_2_SO_4_ (96%) in the presence of carborundum beads. After digestion, the ascorbic acid method was followed to measure P in triplicate from an appropriately diluted solution. Ascorbic acid and Sb were added to the yellow-colored complex solution to convert a blue-colored phosphomolybdenum complex. The method described by Sarker and Oba^25,35^ was followed to read the absorbance by atomic absorption spectrophotometry (AAS) (Hitachi, Tokyo, Japan) at wavelength of 285.2 nm (magnesium), 76 6.5 nm (potassium), 248.3 nm (iron), 422.7 nm (calcium), 279.5 nm (manganese), 213.9 nm (zinc), 324.8 nm (copper).

### Estimation of carotenoids and chlorophylls

Method of Sarker and Oba^35,37^ was followed to estimate chlorophyll *ab*, chlorophyll *b*, total carotenoids, and chlorophyll *a* through extracting the fresh leaves of *A. blitum* in 80% acetone. The absorbance was read at 663 nm for chlorophyll *a*, 646 nm for chlorophyll *b*, and 470 nm for total carotenoids, respectively using a spectrophotometer (Hitachi, U-1800, Tokyo, Japan). Data were expressed as mg chlorophyll per 100 g and µg total carotenoids per g fresh weight.

### Estimation of betacyanin and betaxanthin composition

Method of Sarker and Oba^35,38^ was followed to estimate betacyanin and betaxanthin through extracting the leaves of *A. blitum* in 80% methyl alcohol having 50 mM ascorbate. Betacyanin and betaxanthin were estimated using a spectrophotometer (Hitachi, U-1800, Tokyo, Japan) at 540 nm for betacyanin and 475 nm for betaxanthin, respectively. The results were expressed as microgram betanin equivalent per 100 gram fresh weight (FW) for betacyanin and micrograms indicaxanthin equivalent per 100 gram FW for betaxanthin.

## Estimation of β-carotene

β**-**carotene content was extracted following the method of Sarker and Oba^35^. Exactly 500 mg of fresh leaf sample was ground thoroughly in a mortar and pestle with 10 ml of 80% acetone. After removing the supernatant in a volumetric flask, the extract was centrifuged at 10,000 × g for 3–4 min. The final volume was brought up to 20 ml. The absorbance was taken at 510 nm and 480 nm using a spectrophotometer (Hitachi, U-1800, Tokyo, Japan). Data were expressed as µg β-carotene per g fresh weight.

The following formula was used to estimate the β-carotene content:

$$\begin{array}{ccl}\beta -carotene & = & 7.6(Abs.\,at\,480)-1.49(Abs.\,at\,510)\\  &  & \times \,Final\,volume/(1000\times fresh\,weight\,of\,leaf\,taken).\end{array}$$

### Vitamin C determination

A spectrophotometer (Hitachi, U-1800, Tokyo, Japan) was used to estimate dehydroascorbic acid (DHA) and ascorbate (AsA) acid from the fresh *A. blitum* leaves. Dithiothreitol (DTT) was used for the pre-incubation of the sample and reduction of DHA into AsA. AsA reduced Fe_3_^+^ to Fe_2_^+^. AsA was estimated through measuring Fe_2_^+^ complexes with 2, 2-dipyridyl^35,39^. Finally, the absorbance of the sample solution was read at 525 nm using a spectrophotometer (Hitachi, U-1800, Tokyo, Japan) and data were expressed as µg vitamin C per g fresh weight. The solution was read at 525 nm and data were expressed as µg vitamin C per g fresh weight.

### Extraction of samples for TAC, TFC, and TPC analysis

The leaf samples were dried in the air in a shade for chemical analysis. Exactly 1 g of grounded dried leaves was extracted in 40 ml of 90% aqueous methanol in a tightly capped bottle (100 ml). We placed the bottles in a shaking water bath (Thomastant T-N22S, Thomas Kagaku Co. Ltd., Japan) for 1 h. The extract was filtered for measuring total antioxidant capacity, flavonoids, and polyphenols.

### Total polyphenols estimation

The method described by Sarker and Oba^35,40^ was followed to estimate the total phenolic content of *A. blitum* leaf samples. The gallic acid was used as a standard phenolic compound. We diluted the Folin-ciocalteu reagent in the ratio of 1:4, reagent: distilled water. Exactly 1 ml Na_2_CO_3_ (10%), and 1 ml diluted folin-ciocalteu solution were added to a test tube containing 50 µl extract and mixed thoroughly for 3 min. The tube was allowed to stand for 1 h in the dark. The absorbance was read at 760 nm using a Hitachi U1800 spectrophotometer (Hitachi, Tokyo, Japan). A standard gallic acid graph was made to determine the concentration of phenolics in the extracts. The results are expressed as μg gallic acid equivalent (GAE) g^−1^ DW.

### Estimation of total flavonoids

The total flavonoid content of *A. blitum* extract was estimated following the AlCl_3_ colorimetric method^35,41^. Exactly 1.5 ml methanol, 0.1 ml 1 M potassium acetate, 0.1 ml 10% aluminum chloride, and 2.8 ml distilled water was added to a test tube containing 500 µl leaf extract and allowed to stand for 30 min at room temperature. The absorbance of the reaction mixture was taken at 415 nm using a Hitachi U1800 spectrophotometer (Hitachi, Tokyo, Japan). TFC is expressed as μg rutin equivalent (RE) g^−1^ dry weight (DW) using rutin as the standard compound.

### Estimation of total antioxidant capacity (TAC)

The antioxidant capacity was determined through diphenyl-picrylhydrazyl (DPPH) radical degradation method^35^. Exactly 1 ml 250 µM DPPH solution was added to a test tube containing 10 µl of leaf extract (in triplicate) with 4 ml distilled water and allowed to stand for 30 min in the dark. The absorbance was read at 517 nm using a Hitachi U1800 spectrophotometer (Hitachi, Tokyo, Japan). The method described by Sarker and Oba^35^ was followed to estimate TAC (ABTS^+^) assay. Exactly 2.6 mM potassium persulfate and 7.4 mM ABTS^+^ solution were used in the stock solutions. For the preparation of the working solution, the two stock solutions were mixed in equal quantities and allowed them to react for 12 h at room temperature in the dark. Exactly 150 μl sample of leaf extract was mixed with 2850 μl of ABTS^+^ solution (1 ml ABTS^+^ solution mixed with 60 ml methanol) and allowed to react for 2 h in the dark. The absorbance was read at 734 nm using a Hitachi U1800 spectrophotometer (Hitachi, Tokyo, Japan) against methanol. The percent of inhibition of DPPH and ABTS^+^ relative to the control were used to determine antioxidant activity using the following equation:

$$Antioxidant\,activity( \% )=(Abs.blank-Abs.sample/Abs.blank)\times 100$$

Where, Abs. blank is the absorbance of the control reaction [10 µl methanol for TAC (DPPH), 150 μl methanol for TAC (ABTS^+^) instead of leaf extract] and Abs. sample is the absorbance of the test compound. Trolox was used as the reference standard, and the results were expressed as μg Trolox equivalent g^−1^ DW.

### Statistical analysis

Mineral, phytopigments, chlorophylls, carotenoids, beta-carotene, vitamin C, polyphenols, flavonoids, and antioxidant activity (ABTS^+^ & DPPH) analysis were evaluated in three independent samples per replication (each sample was prepared from a combined sample of leaves from multiple plants) and nine samples per genotype^41^. Results were expressed as mean value ± standard deviation per genotype. Every mean represents the average of all measurements for the same genotype (Tables 1–4). ANOVA was performed using Statistix 8 software and the means were compared by Tukey’s HSD test at 1% and level of probability.

### Ethical statement

The lab and field experiments in this study were carried out as per guidelines and recommendations of “Biosafety Guidelines of Bangladesh” published by the Ministry of Environment and Forest, Government of the People’s Republic of Bangladesh (2005).

## Data Availability

Data used in this manuscript will be available to the public.
